# New insights into the natural history of bronchopulmonary dysplasia from proteomics and multiplexed immunohistochemistry

**DOI:** 10.1152/ajplung.00130.2023

**Published:** 2023-07-25

**Authors:** Andrew M. Dylag, Ravi S. Misra, Gautam Bandyopadhyay, Cory Poole, Heidie L. Huyck, Matthew G. Jehrio, Jeannie Haak, Gail H. Deutsch, Carly Dvorak, Heather M. Olson, Vanessa Paurus, Philip J. Katzman, Jongmin Woo, Jeffrey M. Purkerson, Joshua N. Adkins, Thomas J. Mariani, Geremy C. Clair, Gloria S. Pryhuber

**Affiliations:** ^1^Department of Pediatrics, University of Rochester Medical Center, Rochester, New York, United States; ^2^Department of Laboratory Medicine and Pathology, University of Washington, University of Washington, Seattle, Washington, United States; ^3^Pacific Northwest National Laboratories, Richland, Washington, United States; ^4^Department of Pathology, University of Rochester Medical Center, Rochester, New York, United States

**Keywords:** bronchopulmonary dysplasia, ferroptosis, immunohistochemistry, proteomics

## Abstract

Bronchopulmonary dysplasia (BPD) is a disease of prematurity related to the arrest of normal lung development. The objective of this study was to better understand how proteome modulation and cell-type shifts are noted in BPD pathology. Pediatric human donors aged 1–3 yr were classified based on history of prematurity and histopathology consistent with “healed” BPD (hBPD, *n* = 3) and “established” BPD (eBPD, *n* = 3) compared with respective full-term born (*n* = 6) age-matched term controls. Proteins were quantified by tandem mass spectroscopy with selected Western blot validations. Multiplexed immunofluorescence (MxIF) microscopy was performed on lung sections to enumerate cell types. Protein abundances and MxIF cell frequencies were compared among groups using ANOVA. Cell type and ontology enrichment were performed using an in-house tool and/or EnrichR. Proteomics detected 5,746 unique proteins, 186 upregulated and 534 downregulated, in eBPD versus control with fewer proteins differentially abundant in hBPD as compared with age-matched term controls. Cell-type enrichment suggested a loss of alveolar type I, alveolar type II, endothelial/capillary, and lymphatics, and an increase in smooth muscle and fibroblasts consistent with MxIF. Histochemistry and Western analysis also supported predictions of upregulated ferroptosis in eBPD versus control. Finally, several extracellular matrix components mapping to angiogenesis signaling pathways were altered in eBPD. Despite clear parsing by protein abundance, comparative MxIF analysis confirms phenotypic variability in BPD. This work provides the first demonstration of tandem mass spectrometry and multiplexed molecular analysis of human lung tissue for critical elucidation of BPD trajectory-defining factors into early childhood.

**NEW & NOTEWORTHY** We provide new insights into the natural history of bronchopulmonary dysplasia in donor human lungs after the neonatal intensive care unit hospitalization. This study provides new insights into how the proteome and histopathology of BPD changes in early childhood, uncovering novel pathways for future study.

## Graphical abstract

**Figure:**
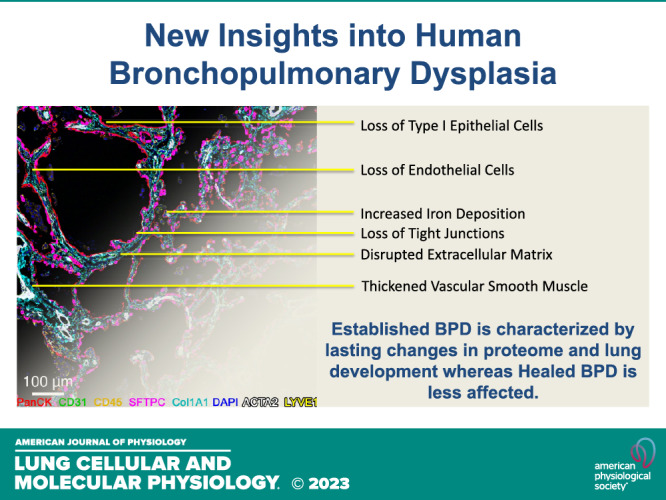

## INTRODUCTION

Bronchopulmonary dysplasia (BPD) is a multifactorial disease of prematurity primarily characterized by arrested lung development and alveolar simplification. Children with BPD suffer from long-term pulmonary morbidity, increased hospitalizations, susceptibility to respiratory viral infections, and neurodevelopmental delays during early childhood and beyond (1–8). Clinical definitions of BPD consider the requirement for respiratory support near-term corrected age (9, 10) with recurrent updates to the definition responsive to criteria that predict longer-term respiratory morbidity (11) but these definitions fail to take into account disease trajectory. Furthermore, BPD is a heterogeneous disease, and the clinical BPD definition often poorly correlates with the histopathologic diagnosis. Some former preterm infants clinically avoid or recover from neonatal lung injury; however, by pathology, they have features of “healed BPD” (hBPD) with alveolar simplification and arrested lung development on lung pathology as first described by Jobe (12). It is unclear if hBPD reflects a truly healed but structurally abnormal lung, or if cellular functions and proteins remain abnormal hence potentially susceptible to therapeutic interventions. More severely affected infants with “established BPD” (eBPD) suffer clinically from ongoing lung injury or failure to repair, leading to superimposed pulmonary hypertension and/or fibrosis. With actionable implications for morbidity, mortality, and therapies, there is a need to determine the degree to which the molecular signatures of hBPD and eBPD evolve after the neonatal intensive care unit (NICU) hospitalization.

Proteomic analyses have been performed on other biologically accessible specimens of human premature neonates such as plasma (13), urine (14), and tracheal aspirates (15). These studies, while minimally invasive, were performed during the neonatal hospitalization early in the natural history of BPD, and only evaluated a limited number of proteins. Given the understandable lack of access to human lung tissues, investigators have turned to proteomic analyses of animal hyperoxia BPD models (16). These models are useful but differ in dose, duration, species, and strain (17, 18) and consider hyperoxia as a singular exposure without the influence of other NICU exposures (i.e., mechanical ventilation, airway organisms, abnormal nutrition). Furthermore, most animal studies are short-term and performed during oxygen exposure disregarding longer-term implications of neonatal hyperoxia with age.

To narrow this knowledge gap, we performed proteomic, molecular, and immunohistochemical analyses of human donor tissues, placed in repository by the lung molecular atlas program (LungMAP) consortium, recovered from term born (“controls”), hBPD, and eBPD subjects who died in early childhood to uncover pathways defining these phenotypes. We hypothesized that hBPD and eBPD would share some similar features (alveolar simplification and loss of pulmonary vasculature) compared with controls, whereas eBPD would demonstrate additional features consistent with ongoing lung injury and cardiopulmonary sequelae of preterm birth.

## METHODS

### Donors and Tissue Procurement

The studied lung samples were authorized by written informed consent of next of kin for donation for research through the United Network of Organ Sharing (UNOS) in coordination with National Disease Research Interchange (NDRI) and International Institute for Advancement of Medicine (IIAM). The University of Rochester IRB approved and oversees the placement and management of these samples in the Biorepository for Investigation of Diseases of the Lung (BRINDL) (RSRB00047606). Criteria for donor acceptance (19) and protocols for whole lung and lobe processing (20) and tissue embedding (21) are available at protocols.io. Samples of ∼0.5 cm^3^ from a standard region of the right middle lobe were flash frozen and stored at −80°C before homogenization for Western blot assay or shipment on dry ice for protein extraction and proteomic analysis. The right lower lobes of each case were inflated with 10% buffered formalin to 20 cmH_2_O, fixed for 24 h, sliced, blocked, and then fixed for an additional 24 h before dehydration to 70% ethanol. The processing into paraffin was done in a standardized fashion with the following protocol available online (22). Tissue sections (4–5 μm) from these formalin fixed paraffin embedded (FFPE) blocks, selected to provide comparable bronchiolar and alveolar regions, were placed on 0.1% poly-l-lysine-coated glass coverslips or Superfrost Plus Microscope Slides (Thermo Fisher Scientific, Waltham, MA) and baked at 50°C for 30 min to enhance slide adherence.

### Protein Extraction and Sample Preparation for Proteomics Analysis

All chemicals for the proteomics sample preparation were obtained from Sigma-Aldrich (St. Louis, MO) with the following exceptions: dithiothreitol, bicinchoninic acid protein assay (BCA), iodoacetamide, and TMTpro 16plex label reagent set were obtained from Thermo Fisher Scientific (Waltham, MA). Trypsin was obtained from USB (Thermo Fisher Scientific). Ultrapurified water was obtained from a Millipore Milli-Q system (St. Louis, MO). A chloroform:methanol extraction (23) was performed on pieces of lung tissue. Briefly, 940 µL of ice-cold 3:4 methanol:water was added to each sample in a 5.0 mL Eppendorf tube (Enfield, CT), and the sample was thoroughly homogenized using an Omni tissue homogenizer (Omni, Kennesaw, GA) with a disposable tip. Following homogenization, 1,060 µL of ice-cold chloroform was added to each sample, and the tubes were vortexed for 1 min. All samples were placed on ice for 5 min followed by another 1-min vortex. The samples were then centrifuged at 10,000 *g* for 10 min at 4°C. The top (polar) layer was transferred to a preweighed automatic liquid sampler (ALS) vial (MicroSolv, Leland, NC), whereas the lower (nonpolar) layer was transferred to a different preweighed ALS vial (Waters Corp., Milford, MA). Both the polar and nonpolar layers were dried completely and then reweighed to obtain the mass of metabolite. The nonpolar samples had 500 µL of 2:1 chloroform:methanol added to each vial and both sets of samples were stored at −20°C until needed for analysis. The precipitated protein interphase was washed with 1 mL of ice-cold methanol, transferred to a 1.5 mL microcentrifuge tube, and centrifuged at 15,000 *g* for 10 min at 4°C. The methanol was decanted, and the pellet was allowed to dry by inverting the tube over a kimwipe for 5 min. Denaturing buffer (8 M urea in 50 mM ammonium bicarbonate) was added to each protein pellet. The pellets were reconstituted to a soluble homogenate using vortexing and brief sonication, then a BCA protein assay was performed to determine the approximate protein concentration of each sample. Protein (500 µg) was utilized from each sample, with volumes normalized using denaturing buffer. Dithiothreitol was added to a 5 mM concentration, and the samples were incubated on a Thermomixer (Eppendorf) for 1 h at 37°C with shaking at 850 rpm. Iodoacetamide was added to a 40 mM concentration, and the samples were incubated as before but in darkness. Samples were diluted eightfold with 1.14 mM CaCl_2_ in 50 mM ammonium bicarbonate, and trypsin was added in a 1:50 (trypsin:protein, wt:wt) ratio. The samples were incubated for 3 h at 37°C with shaking at 850 rpm, then frozen and stored at −70°C. Solid phase extraction (SPE) was performed on the samples using 1 mL/50 mg C18 columns from Phenomenex (Torrance, CA). Columns were conditioned with 2 mL of methanol followed by 3 mL of 0.1% trifluoroacetic acid (TFA) in water. A sample was applied to each column followed by 4 mL of 95:4.9:0.1 H_2_O:MeCN:TFA. Samples were eluted into 1.5 mL microcentrifuge tubes with 80:19.9:0.1 MeCN:H2O:TFA and concentrated in a vacuum concentrator to 50 µL. Another BCA protein assay was performed to obtain peptide concentration and a portion of each sample was diluted to 0.1 µg/µL for LC-MS analysis. For tandem mass tag (TMT) labeling, 100 µg of peptides from each sample were aliquoted, dried, and reconstituted to a 5 µg/µL concentration with 50 mM HEPES, pH 8.5. TMT reagent (20 mg/mL) was added to the peptides in a 1:1 ratio (wt:wt). Samples were incubated at 25°C for 1 h at 400 rpm in a Thermomixer. Samples were then diluted to 2.5 µg/µL with 50 mM HEPES pH 8.5, 20% acetonitrile. The samples labeled with different TMT reagents were combined into one tube, diluted with water to reduce the acetonitrile concentration to less than 5%, and desalted using C18 solid phase extraction (SPE). The multiplexed sample was concentrated to 100 µL, and a BCA protein assay was again performed for quantitation. The peptide mixture was then fractionated using high pH reverse phase chromatography on a Waters XBridge column and concatenated into 24 fractions as outlined in Wang et al. (24). Each fraction was concentrated on a vacuum concentrator to 50 µL, the peptides were then quantitated and diluted with water to 0.07 µg/µL for LC-MS analysis.

### LC-MS/MS Proteomics

The LC was configured to load the sample first on an SPE column followed by separation on an analytical column. Analytical columns were made in-house by slurry packing 3 μm Jupiter C18 stationary phase (Phenomenex) into a 70 cm long, 360 μm OD × 75 μm ID fused silica capillary tubing (Polymicro Technologies). Samples were loaded on the SPE column via a 5 μL sample loop for 30 min at a flow rate of 5 μL/min and then separated by the analytical column using a 140 min gradient from 99% mobile phase A (MP-A) to 5% MP-A at a flow rate of 300 nL/min. MS analysis was started 20 min after the sample was moved to the analytical column. After the gradient was completed, column was washed with 100% mobile phase B (MP-B) first and then reconditioned with 99% MP-A for 30 min. The effluents from the LC column were ionized by electrospray ionization by applying 2,200 V to the metal union between the column and the electrospray tip. Electrosprayed ions were introduced into the mass spectrometer (Orbitrap Q Exactive Plus) via a heated capillary (5.8 cm long with a rectangular slit of 1.6 mm long and 0.6 mm wide) maintained at 300°C for ion desolvation. The resulting ions were mass analyzed by the Orbitrap at a resolution of 70,000 covering the mass range from 300 to 2,000 Da with a maximum injection time of 50 ms and automated gain control (AGC) setting of 3E6 ions. Mass spectra were recorded in profile mode. Most abundant ions were subjected to MS2 analysis using the top speed mode, acquiring the top 12 ions in the cycle time. The parameters used for these analyses were as follows. For MS2, ions were isolated by quadrupole mass filter in monoisotopic peak selection mode using isolation window of 0.7 Da, maximum injection time of 100 ms with AGC setting at 5E4 ions, and fragmented by high-energy collision dissociation (HCD) with nitrogen at 30% normalized collision energy. Fragment ions were mass analyzed by the Orbitrap at a resolution of 35,000, and spectra were recorded in the centroid mode. Ions once selected for MS2 were dynamically excluded for the next 30 s. Mass spectra were recorded in centroid mode. The instrument raw files are publicly available on MassIVE (Server: massive.ucsd.edu, User: MSV000091493).

### Proteomics Data Analysis

Raw MS data were analyzed using MaxQuant 2.1.4.0 against Uniprot redundant Homo sapiens database (79,739 sequences downloaded 10/10/2022) and contaminants. Two unique peptides were required and false discovery rate (FDR) was set to 1% at the spectral, peptide, and protein levels. Statistical analyses were performed using in-house RomicsProcessor v1.1.0 (R package, https://github. com/PNNL-Comp-Mass-Spec/RomicsProcessor). The data analysis code is publicly available at https://github.com/GeremyClair/Proteome_analysis_of_a_human_donor_cohort_to_study_bronchiopulmonary_displasia.

Briefly, the data were imported as a multilayered R object with its associated metadata. The intensities were then log2 transformed and filtered to allow maximal missingness of 70% within at least one given group. After median normalization, the missing values were imputed using a previously described method using a random downshifted distribution (25). A principal component analysis (PCA)-based dimension reduction analysis was performed. ANOVA was used to compare across experimental groups (control, hBPD, and eBPD), and post hoc Student’s *t* tests were performed to compare the different groups. As the main differences were observed between control and eBPD, *t* test was also systematically performed between these two groups. Enrichment analyses were performed using in-house R package Protein-MiniOn v1.0 using the same Fisher’s exact modified EASE test as the DAVID bioinformatic resource (26). Modulated proteins found in the matrisomeDB (27) were extracted from the main table to determine the extracellular matrix proteins altered in the study samples.

### Multiplexed Immunohistochemistry

Lung tissue sections on l-lysine-coated coverslips were deparaffinized in xylene (3 × 5 min), cleared in 100% ethanol, and rehydrated via a decreasing ethanol series. Antigen retrieval was performed by immersion in 1× antigen unmasking solution, citrate based (H-3300, Vector Laboratories Inc., Newark, CA) and heated in an Instant Pot for 20 min. After cooling, the lung tissue was washed two times for 2 min with hydration buffer (Akoya Biosciences, Marlborough, MA) and then incubated for 20–30 min in staining buffer (Akoya). Coverslips were then covered with 200 μL of antibody buffer composed of staining buffer supplemented with recommended concentrations of N, G, J, and S blockers (Akoya), the specified dilutions of 18 antibodies and their respective bar codes that comprise the BPD Codex panel (Supplemental Table S1) and incubated in a humidified chamber for 3 h. Sections were then washed twice for 2 min with staining buffer followed by fixation in 1.6% paraformaldehyde for 10 min. Sections were rinsed in PBS (3 × 3 dips each) before fixation in ice-cold methanol for 5 min. Following a PBS rinse (3 × 3 dips), sections were covered with a 1:50 dilution of final fixation solution (Akoya) and incubated for 20 min in a humidified chamber. Sections were rinsed in PBS (3 × 3 dips) before placing in 5 mL storage buffer (Akoya) and photobleached by illumination with a 200 mA, 15 W, 1,600 lumens bulb overnight at 4°C. Coverslips were mounted on a stage insert and initially stained with DAPI (14.3 µM) for 3–5 min to facilitate the identification of a region of interest (ROI) using a Keyence BZ-X810 inverted epifluorescence microscope equipped with: *1*) Keyence Pan Fluorite 4X/0.13 NA and Nikon 20x Plan Fluorite 0.75 NA objectives, *2*) four filter cubes including CH1: DAPI (Ex 350, Em 461), CH2: Cy3/R ATT0550 (Ex 545, Em 605), CH3: Cy5 AF633(Ex 632, Em 647), CH4: Cy7 AF750 (Ex 743, Em 767), and interfaced with a Phenocycler (when formerly CODEX) fluidics system (Akoya). Once an ROI was identified with the ×4 objective, 10–30 *Z*-plane stacks within the ROI were imaged with the ×20 objective over eight cycles with the indicated reporters and exposure times for respective channels (Supplemental Table S2). Processing (i.e., background subtraction, deconvolution, extended depth of field, shading correction, full stitching, and diagnostic output) of *Z*-plane tile stacks was performed with CODEX Processor 1.8.3.14 software (Akoya).

### Imaging Analyses

Processed and stitched images for the respective cycle and up to seven channels (Supplemental Table S1) were merged, for human color perception, utilizing ImageJ (Fiji) (28). Alternatively, greater than seven channels were merged utilizing an open source ImageCombiner extension in QuPath bioimage analysis software (29). Merged images were analyzed in QuPath as pyramidal fluorescent images. Segmentation of lung bronchiole and vascular structures, as well as alveolar space, was performed utilizing the wand and brush tools in QuPath. Collagen type 1 alpha 1 chain (COL1A1), smooth muscle actin alpha 2 (ACTA2), and lymphatic vessel endothelial hyaluronan receptor 1 (LYVE1) expression in segmented alveolar space was determined by pixel classification based on thresholding signal intensity of the respective channel from which the percent signal area with respect to the alveolar area was determined. The cell detection algorithm in QuPath was used to identify nuclei [Detection Channel = DAPI; Nucleus Parameters (px): Background Radius = 15; Median Filter Radius = 5; Sigma = 3; Min. area = 10–20; Max area = 1,000–2,000. Intensity Parameters: Threshold = 300–3,000; split by shape. Cell Parameters: Cell Expansion = 5–10; split by shape]. Cell classifiers were then devised by thresholding the signal intensity for the respective cell markers (Supplemental Table S1), thereby enabling enumeration of epithelial and immune cell phenotypes utilizing single or composite phenotypic markers. Relative cell numbers between images were normalized to the respective segmented area. Statistical significance between groups was determined by one-tailed *t* test (*P* < 0.05).

### Bright-field Immunohistochemistry

#### Hematoxylin and eosin.

FFPE sections (5 µm) were stained by standard hematoxylin-eosin (H&E) methods, and the protocol is available online at protocols.io (30). Whole slide bright-field images were from tiles captured with the Keyence BZ-X810 microscope, transferred, and visualized in Omero.web v. 5.8.1 (31).

#### Prussian blue.

FFPE sections were deparaffinized with xylene and rehydrated to PBS with decreasing amounts of ethanol. They were immersed in 20% hydrochloric acid (Thermo Fisher Scientific, Waltham, MA):10% potassium ferrocyanide (Sigma-Aldrich, St. Louis, MO) solution for 20 min to stain ferric iron bright blue. They were then counterstained with nuclear fast red (Sigma-Aldrich) for 5 min to stain nuclei red and cytoplasm pink. Slides were dehydrated in increasing amounts of ethanol, cleared with xylene, and mounted with toluene-based media (Permount, Thermo Fisher Scientific).

### Quantitation of Vascular Smooth Muscle Wall Thickness in Whole Slide Images

Smooth muscle actin was stained after deparaffinization and rehydration of 4-µm thick FFPE sections using a Dako Target Retrieval Solution (pH 9, 10×, at 98°C, for 35 min). Sections were blocked with 10% normal goat serum (NGS) in Tris-buffered saline (TBS)-Tween (0.05%) for 60 min and incubated with mouse monoclonal anti-ACTA2 (1:2,000, clone 1A4, Sigma-Aldrich) diluted in a 5% normal goat serum overnight at 4°C. The sections were then washed and stained with a secondary antibody [Alexa 594-conjugated goat anti-mouse IgG2a, 1:200 in Tris-buffered saline-Tween 20 (TBST), 60 min, RT] followed by a counterstain with DAPI (14.3 µM) in TBS for 10 min. All slides were coverslipped with ProLong Glass mounting medium (Invitrogen).

Whole slide image fields (∼1 cm^2^) were created from tiled images captured with a Keyence BZ-X810 inverted epifluorescence microscope equipped with a ×10 Plan Fluorite 0.3 NA lens and standard DAPI and mCherry cubes (Exposure 1/8.5–1/15 s), or sans cube for bright-field imaging, and postprocessing with the Microscopy Image Stitching Tool (MIST) (32). ACTA2 staining in vasculature was quantitated utilizing QuPath by training the pixel classifier [Artificial neural network (ANN_MLP), Full Resolution (downsample = 1.0), minimum object size: 3,000 px^2^, minimum hole size 300–400 px^2^] on five to seven segmented vascular structures in representative images. The pixel classifier was trained to ignore ACTA2 staining associated within bronchioles. Vascular smooth muscle (VSM) thickness was quantitated as area (px^2^)/perimeter (px) of segmented ACTA2 staining. The percentage of cells positive for Prussian blue staining in whole slide images (WSI) was determined utilizing the cell detection algorithm in QuPath to detect all cells by thresholding nuclear fast red stain [Detection Image= Optical Density sum; requested pixel size = 0.5; Nucleus Parameters (px): Background Radius = 8; Median Filter Radius = 0; Sigma = 1.5; Min. area = 5; Max area = 400. Intensity Parameters: Threshold = 0.02–0.1; Max BKGD Intensity = 2; split by shape. Cell Parameters: Cell Expansion = 2.5–5 µM; split by shape; include cell nucleus; General Parameters: smooth boundaries; make measurements] followed by detection of Prussian blue positive cells by thresholding blue stain [Detection Image = Hematoxylin OD; requested pixel size = 0.5; Nucleus Parameters (px): Background Radius = 8; Median Filter Radius = 0; Sigma = 1.5; Min. area = 10; Max area = 400. Intensity Parameters: Threshold = 0.1; Max BKGD Intensity = 2; split by shape. Cell Parameters: Cell Expansion = 5 µM; split by shape; include cell nucleus; General Parameters: smooth boundaries; make measurements.]

### Western Blots

BRINDL lung samples, ∼0.5 cm^3^, were maintained at −80°C until homogenized in protein lysis buffer (50 mmol/L Tris, pH 7.4, 150 mmol/L sodium chloride, 2 mmol/L ethylenediamine tetraacetic acid, 25 mmol/L sodium fluoride, ß-glycerolphosphate, 0.1 mmol/L sodium vanadate, 1 mmol/L phenylmethyl sulfonyl fluoride, 0.2% Triton X-100, 0.3% Igepal CA-630, 10 g/mL leupeptin, 10 g/mL pepstatin A, 10 g/mL aprotinin; Sigma Chemical Co.). Total protein content of the lysates was determined by bicinchoninic acid assay using commercial reagents and protocol (Pierce, Inc., Rockford, IL). Samples in Laemmli buffer were size separated on 15% polyacrylamide-sodium dodecyl sulfate resolving gel with 5% stacking gel before transfer to a PVDF membrane (IPVH00010, Millipore, Darmstadt, Germany). Membranes were then blocked in 5% nonfat milk in TBS-T, stained with primary antibodies [Anti-FTL (1:1,000, ab218400, mouse monoclonal, Abcam, Cambridge, UK) and Anti-FTH1 (1:500, ab75972, rabbit monoclonal, Abcam)] and secondary antibodies [FTL: anti-mouse IgG(H + L), 1031-05, SouthernBiotech, Birmingham, AL; FTH1: anti-rabbit IgG(H + L), Mouse/Human ads-HRP, 4050-05, SouthernBiotech]. Anti-GAPDH primary (1:1,000, AF5718, R&D, Minneapolis, MN) and secondary (6160-05, SouthernBiotech) antibodies were used as an internal loading control. Immunodetection was performed using enhanced chemiluminescence protocols and reagents (ECL Plus; Amersham, Arlington Heights, IL). Blots were measured using ImageJ (NIH, Bethesda, MD), with each isoform measured separately, and data were analyzed with Graph Pad Prism 9 (San Diego, CA) using one-tailed *t* tests.

## RESULTS

### Cohort Description

Pediatric human donors were classified based on postnatal age and perinatal history, confirmed by histopathology (Supplemental Fig. S1), as “healed” BPD (hBPD, 3 yr of age, *n* = 3), “established” BPD (eBPD, 1 yr of age, *n* = 3), or as corrected age-matched controls (*n* = 6) with no known lung disease (Table 1). All BPD subjects had a history of premature birth (23–26 wk’ gestational age), whereas the control subjects were born at term. There was no difference between the gestational age at birth of those with eBPD as compared with hBPD. LungMAP human tissue core protocols for pathologist’s review found all control lungs to have normal growth and development, whereas hBPD and eBPD both displayed alveolar simplification with reduced alveolar count for age, corrected for preterm birth (33), and in eBPD the additional presence of disruption of the underlying lung architecture, including interstitial fibrosis and increased smooth muscle in the lobule, was consistent with previously described stages of BPD (12, 34).

### The Proteome of eBPD Lungs is Distinct from Controls

**Figure 1.**
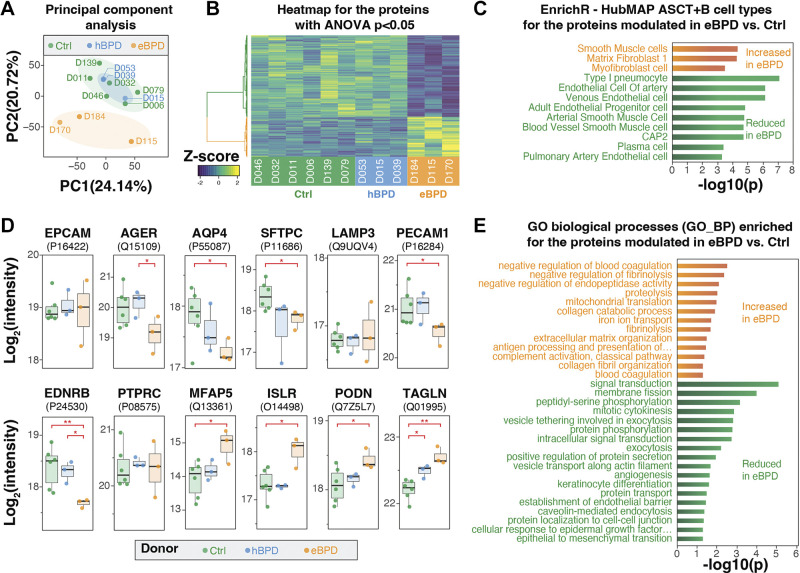
Proteome analysis of BPD donors. *A*: principal component analysis show that the “established” (eBPD) proteome segregate from the “healed” (hBPD) and control (Ctrl) proteome profiles. *B*: heatmap depicting the proteins modulated (ANOVA *P* < 0.05). Protein hierarchical clustering shows two major clusters of protein abundance (Euclidean distance, Ward.d clustering method). *C*: enrichment analysis was performed to identify cell population markers enriched in the comparison eBPD vs. Ctrl on EnrichR against the HubMAP ASCT+B table (all enrichments with *P* < 0.05 depicted). *D*: normalized proteomics abundance for cell population/type markers. Stars indicate *t* test *P* values (*P < 0.05,**P < 0.01) performed between two groups indicated by the red brackets. *E*: enrichment analysis was performed to identify Gene Ontology biological processes modulated in eBPD using Protein Mini-on (enrichments with *P* < 0.05 depicted). ANOVA, analysis of variance; BPD, bronchopulmonary dysplasia.

Proteome analysis of the lungs from different human donors resulted in the identification of 5,739 quantifiable proteins. A principal component analysis (PCA; Fig. 1*A*) indicates that the proteomes of eBPD samples and of the control group clustered separately, whereas the hBPD subjects’ proteome did not segregate from controls on the first two nor on subsequent principal components (e.g., 3–6). An ANOVA was used to identify the 768 differentially abundant proteins (Fig. 1*B*). In most cases, the proteins had similar abundance patterns in the control and hBPD but were modulated in the eBPD group. Therefore, we focused our further analyses on the comparison of the eBPD and the control groups. A Student’s *t* test was used to identify the 186 proteins increased and 534 decreased in abundance in the eBPD group compared with controls (*P* < 0.05).

### Proteomic Profiling Suggests Cell Population Shifts in BPD

We attempted to identify cell types that were modulated from the proteomics data using EnrichR (35) and the HubMAP “Anatomical Structures, Cell Types, plus Biomarkers” table for lung v1.0 available online at HubMAP portal—https://hubmapconsortium.github.io/ccf/pages/ccf-anatomical-structures.html (36). We hypothesized that if we found multiple markers of a given cell type trended similarly in eBPD lungs compared with controls, it could indicate the modulation of the count of this cell type. EnrichR suggested that type I pneumocytes (AT1) and endothelial cells were the most depleted cell populations in eBPD, whereas some smooth muscle cell and fibroblast populations increase (Fig. 1*C*). To further explore changes that may occur in cell populations, we plotted the abundance of select markers for cell types and pan-populations (Fig. 1*D*). Epithelial cell adhesion molecule (EPCAM), a canonical pan-epithelial marker (37, 38), was not modulated between the eBPD and control groups. However, advanced glycosylation end-product specific receptor (AGER) (39, 40) and aquaporin 4 (AQP4) (41), both alveolar type 1 (AT1) markers, were decreased in the eBPD group. Surfactant protein C (SFTPC), which is known to be almost exclusively expressed by alveolar type 2 (AT2) cells, was reduced in eBPD lungs, whereas lysosomal membrane-associated protein (LAMP3), a marker of dendritic cells and AT2 cells, did not change. Platelet and endothelial cell adhesion molecule 1 (PECAM-1/CD31), a canonical pan-endothelial marker (34, 42), was decreased in the eBPD group suggesting a general reduction of the proportion of endothelial cells. More specifically, endothelin receptor type B (EDNRB), a marker of capillary 2 (Cap2) cells (43–46), was reduced in the eBPD cases (47–49). The fibroblast markers immunoglobulin super family containing leucine-rich repeat protein (ISLR) (48), microfibril associated protein 5 (MFAP5) (45, 50), podocan (PODN) (51), and transgelin (TAGLN), a smooth muscle and/or pericyte marker (45, 49, 52), were all increased in eBPD tissues. Notably, protein tyrosine phosphatase receptor type C (PTPRC/CD45), a pan-immune marker (34, 53), did not change in abundance between control or BPD tissues. Finally, TAGLN was the only marker of this subset increased in hBPD compared with controls, reaffirming that protein differences in hBPD are more limited than in eBPD.

### Functional Proteome Profiling in BPD

A functional enrichment analysis was performed to identify the Gene Ontology (GO) terms associated with the changes in protein abundance in eBPD (Fig. 1*E*). The proteins more abundant in BPD were enriched in the GO terms related to blood coagulation (GO:0007596, GO:0030195), proteolysis (GO:0006508, GO:0010951), complement activation (GO:0006958), antigen processing (GO:0019886), iron-related processes (GO:0006879, GO:0008199), and collagen-related processes (GO:0062023, GO:0030574). The proteins less abundant in BPD were enriched in GO terms related to signal transduction (GO:0007165), tight junctions (GO:0150105, GO:0005925), mitosis (GO:0090148, GO:0000281), protein phosphorylation (GO:0090522, GO:0018105), vesicle trafficking (GO:0090522, GO:0072584), and angiogenesis (GO:0001525, GO:0061028).

### MxIF Supports Changes in Cell Type Abundance in eBPD Lungs

**Figure 2.**
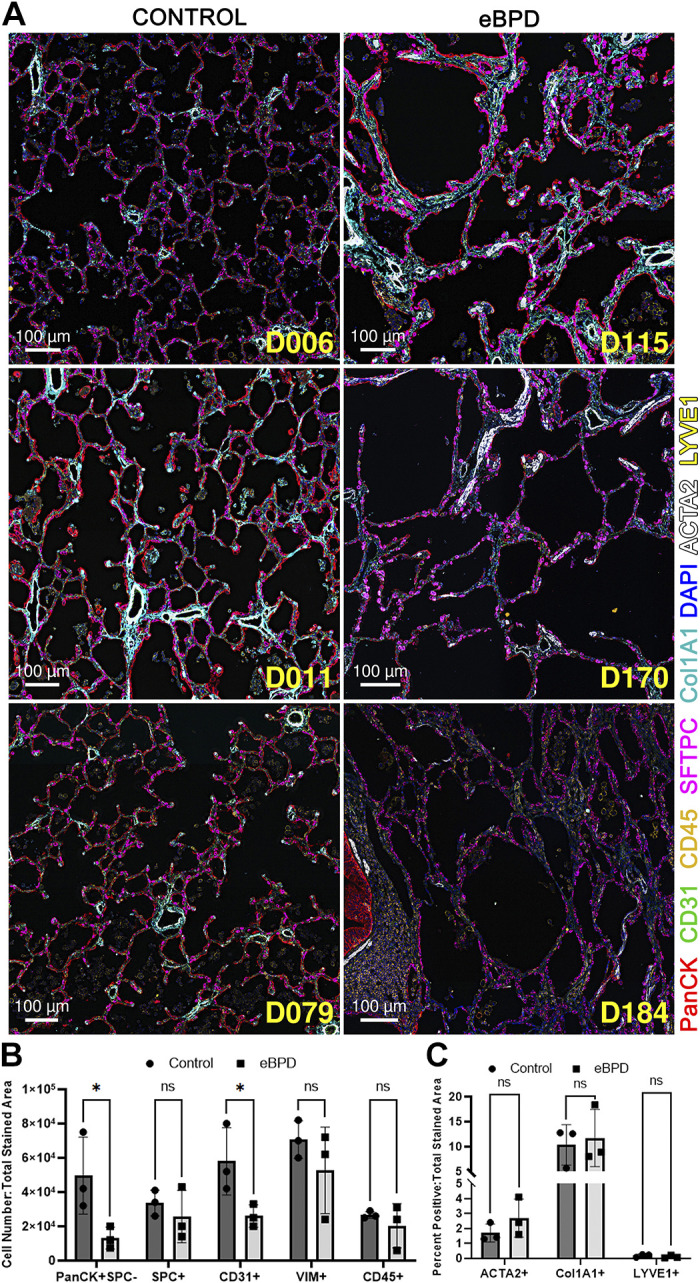
Multiplexed fluorescent imaging reveals epithelial and endothelial cell loss due to BPD-associated alveolar simplification. *A*: lung tissue sections from control (*left*) and eBPD donors (*right*) were stained with an 18-marker panel and a region of interest (ROI) was sampled from the alveolar space (see Supplemental Fig. S4 for digital zoom). Eight markers including PanCK (red), CD31 (green), DAPI (blue), ACTA2 (white), Col1A1 (cyan), CD45, (orange) SFTPC (magenta), and LYVE1 (yellow) are shown. *B*: graphical representation of staining frequency of cell types in highly multiplexed antibody panel, enumerated by cell detection and thresholding of the respective markers(s) utilizing QuPATH image analysis software. The number of pan-cytokeratin (PanCK+SPC−) and CD31+ cells decreased in eBPD. *C*: staining of area for other markers was performed by thresholding in QuPATH in either segmented alveolar area (ACTA2, COL1A1) or the whole ROI image (LYVE1). Differences were not observed in staining area for the above markers. BPD, bronchopulmonary dysplasia; eBPD, established bronchopulmonary dysplasia; SFTPC, surfactant protein C. *P < 0.05.

To link the changes in protein abundance to the gain and loss of lung cell types, we performed multiplexed immunohistochemistry on a panel of 18 proteins in the eBPD donors compared with their age-matched controls (Table 1). Based on antibody staining of one or more panel markers, cells were enumerated based on costaining with DAPI and normalized to the total stained area in the region of interest. Focusing only on alveolar regions, we observed decreased pan-cytokeratin (PanCK)+ surfactant protein C (SPC)- (AT1 cell) staining in eBPD versus control, whereas SPC+ (AT2) cell number was not changed (Fig. 2*B*) suggesting that the loss of epithelial cells was predominantly AT1 cells, consistent with proteomic cell type predictions (Fig. 1, *C* and *D*). Furthermore, CD31+ cells were found in decreased abundance in eBPD lungs compared with control indicating a loss of lung capillary vasculature consistent with alveolar simplification (Fig. 2*B*). Surprisingly, we did not find increased PTPRC/CD45+ cells in the lungs and deeper immune profiling by MxIF showed highly variable numbers of CD3+, CD3+/CD4+, CD20 + HLA-DR (human leukocyte antigen-DR isotype)+, CD68 + HLA-DR+, and CD1c + HLA-DR+ immune cells that did not change in eBPD compared with age-matched controls, though the percent of CD1c cells was higher in eBPD (Supplemental Fig. S2).

**Table 1.** Donor demographics and clinical/pathological assessments

Donor Id	BPD or Control, CNT	PNA, yr	RAC	GA, wk	PMA, wk	Sex	Race/Ethnicity	Health Status	Histo-Pathological Findings
D079	CNT	1.31	9.2	40/U	108.1	M	White	Healthy	Normal growth and development, mild-mod pneumonia
D006	CNT	1.62	11	40/U	124.3	F	White	Healthy	Normal structure and development, patchy mild bronchiolitis and pneumonia
D011	CNT	1.74	8.9	40/U	130.3	F	White	Healthy	Normal structure and development
D184	eBPD	1.27	6.5	24	89.9	M	White	Home vent dependent for BPD, budesonide, levalbuterol	CLD/ new BPD, mod—marked alveolar simplification; periairway and lobular lymphocytic aggregates; mild bronchiectasis; arteriopathy—mild medial hypertrophy, large PAs rare thrombus
D115	eBPD	1.62	*	26	110.3	F	White	Vent dependent, PHTN on VD, ineligible for transplant due to neuro status	Classic CLD/BPD, marked alveolar simplification; lobular fibrosis and smooth muscle hyperplasia; reactive airway epithelium; macrophage accumulation; arteriopathy—PA medial hypertrophy, intimal hyperplasia
D170	eBPD	1.81	4.6	25	119.6	F	Black	Vent dependent, marked inflation right lung, PHTN on VD, left heart diastolic dysfx	CLD/BPD, marked alveolar simplification; patchy necrotizing bronchiolitis; mild arterial medial hypertrophy, PAs; scattered hemosiderin
D046	CNT	3.05	10.6	40/U	198.9	M	White	Healthy	Normal growth and development
D032	CNT	3.53	12.8	40/U	223.6	M	White	Healthy	Normal growth and development
D139	CNT	3.99	9.3	40/U	247.3	F	White	Healthy	Normal lung growth and structure
D053	hBPD	3.16	7.1	24	188.9	M	White	Home, no vent, albuterol PRN, VP shunt malfx	CLD/BPD, moderate deficient lung growth with reduced lung weight and RAC; mild-mod chronic bronchiolitis;
D039	hBPD	3.27	5.8	23	193.6	F	Hispanic	Home, no vent, no resp meds, VP shunt malfx	Classic CLD/ new BPD, mild-mod deficient lung growth esp subpleural; minimal fibrosis; mild acute and chronic airway inflammation; mild PA medial hypertrophy
D015	hBPD	3.89	6.2	25	227.7	F	White	Home, no vent, no resp meds, Neuro event	CLD/BPD, diffuse necrotizing aspiration pneumonia with yeast; deficient lung growth with reduced lung weight and RAC

Age-matched healthy donors and ex-ELGANS with established (eBPD) or healed (hBPD) cases listed by postnatal age in years. BPD, bronchopulmonary dysplasia; CLD, chronic neonatal lung disease; eBPD, established BPD; ELGANS, extremely low gestational age newborns; GA, gestational age at birth; hBPD, healed BPD; PAs, pulmonary arteries; PHTN, pulmonary hypertension; PMA, post menstrual age in weeks corrected for preterm gestational age [PMA in weeks = PNA in weeks + (40 – weeks premature)]; PNA, postnatal age; RAC, radial alveolar count (normal average = 10); U, gestational age not reported, presumed full term; VD, pulmonary vasodilators; VP shunt malfx, ventriculoperitoneal shunt malfunction. *RAC could not be determined due to severe lung remodeling.

### Immunohistochemistry Indicates Increased Vascular Smooth Muscle in eBPD

**Figure 3.**
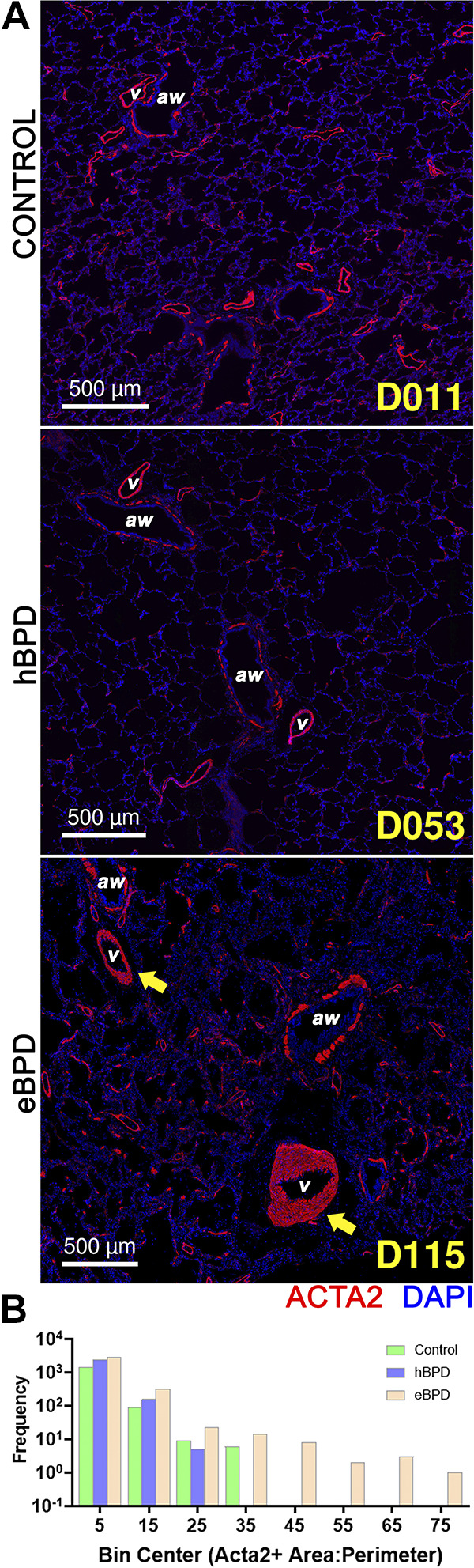
BPD is associated with vascular smooth muscle (VSM) hypertrophy. *A*: representative lung sections from control (*top*), hBPD (*middle*), and eBPD (*bottom*). BPD stained for nuclei (DAPI, blue) and smooth muscle actin (ACTA2, red). Yellow arrows point to VSM hypertrophy in established BPD. *B*: frequency distributions of ACTA2+ staining area:perimeter for whole slide images of lung sections for all donors utilizing QuPath software. Wilcoxon rank-sum tests between each pair of samples indicated significant (*P* < 0.0001) differences in distribution between eBPD and hBPD/controls. BPD, bronchopulmonary dysplasia.

We pursued a deeper histological and spatial analysis of the smooth muscle cell populations in the BPD lung because ACTA2+ cell area staining in the eBPD lungs was not different on a global level (Fig. 2*C*). A significant amount of the ACTA2+ signal can be seen in the smaller airway and parenchymal structures. We analyzed whole slide imaging using machine learning to detect vascular smooth muscle and ignore airway smooth muscle (Fig. 3). To normalize for vessel size, vascular smooth muscle thickness was determined by plotting airway ACTA2+ vessel wall area/perimeter (A/P) with higher ratios indicating a thicker smooth muscle layer. We detected increased ACTA2+ A/P signal in higher perimeter (medium to large) vascular structures in eBPD lungs suggesting increased vascular smooth muscle thickness (*P* < 0.0001 for Kruskal–Wallis test). This is consistent with two of the three eBPD donors having clinical pulmonary hypertension.

### Proteomics Suggests a Reshaping of the Extracellular Matrix in eBPD

**Figure 4.**
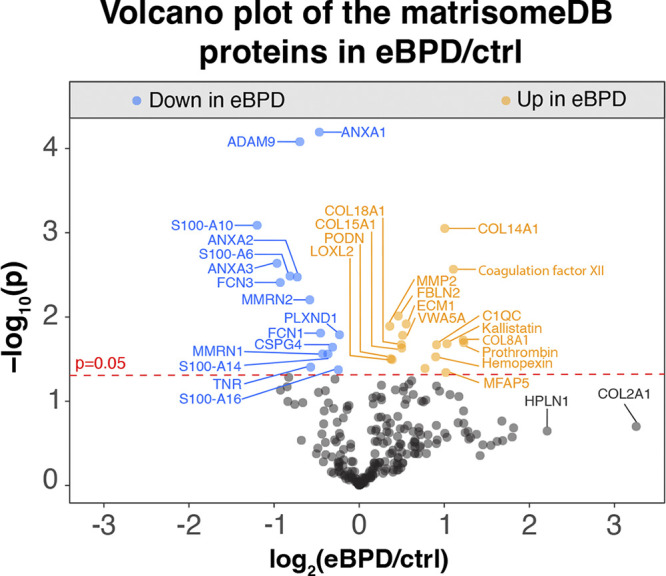
Volcano plot depicting the matrisome proteins modulated in eBPD vs. Ctrl. Student’s *t* test *P* values were used, and protein list was filtered to extracellular matrix proteins (MatrisomeDB.org, v.1.0.). eBPD, established bronchopulmonary dysplasia.

The proteins associated with the extracellular matrix were identified based on MatrisomeDB (54, 55). We were able to quantify 248 MatrisomeDB proteins using the global MPLEx method that uses a chloroform:methanol protein extraction (23). This confirms that the MPLEx method enables the extraction and quantification of extracellular matrix by mass spectrometry as previously shown in the normal developing lung (56). In this analysis, we found 15 core matrisome or matrisome-associated proteins decreased in eBPD compared with controls including multimerins 1 and 2 (MMRN1 and MMRN2), multiple annexins (ANXA1, ANXA2, and ANXA3), and multiple proteins of the S100 family (Fig. 4). Conversely, among 17 proteins increased in eBPD were multiple collagens (COL8A1, COL14A1, COL15A1, and COL18A1) and proteins associated with coagulation (coagulation factor XII, prothrombin, hemopexin; Fig. 4).

### Increased Iron-Related Proteins Are Present in eBPD

**Figure 5.**
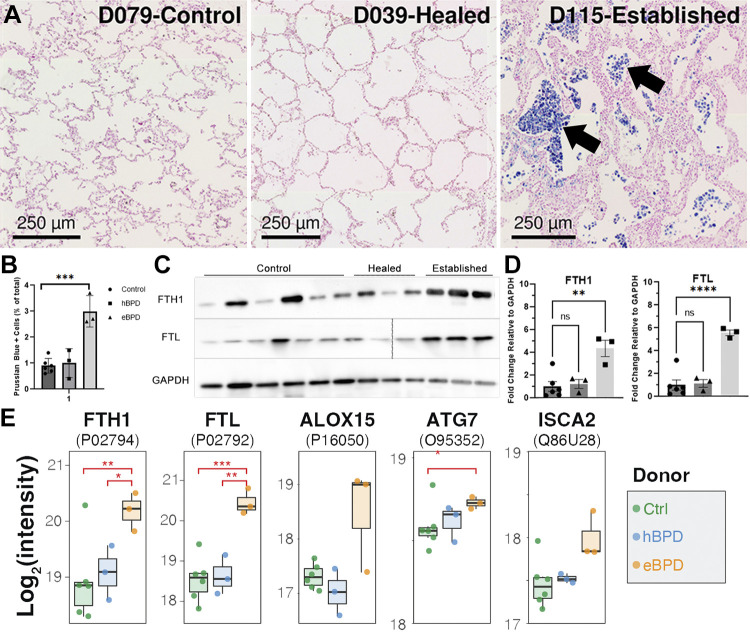
Iron and ferroptosis-related proteins are upregulated in BPD. *A*: representative brightfield Prussian blue staining for iron in whole tissue sections from control (*left*), hBPD (*middle*), and eBPD (*right*). *B*: quantification of Prussian blue+ cells show increased iron deposition in eBPD vs. other groups. *C*: Western blots, control *n* = 6, hBPD *n* = 3, eBPD *n* = 3, (dotted line in FTL blot indicates where empty lane was removed from the image). See Supplemental Fig. S3, *A*–*F*, for labeled unedited blots. *D*: quantification of Western blots for FTH1 (*top*), FTL (*middle*), and GAPDH loading control (*bottom*) for each sample in the experimental groups. *E*: mass spectrometry intensities for iron and ferroptosis-related proteins in the data set. Stars indicate *t* test *P* values (*P < 0.05, **P < 0.01, ***P < 0.001, ****P < 0.0001) performed between groups indicated by brackets. BPD, bronchopulmonary dysplasia; eBPD, established bronchopulmonary dysplasia; hBPD, healed bronchopulmonary dysplasia.

Iron-related processes were enriched in the eBPD group (Fig. 1*E*) that can be linked to ferroptosis. Ferroptosis is a recently described cellular process leading to cell death characterized by cellular iron accumulation and lipid peroxidation (57). During ferroptosis, the unstable iron pool accumulates and can be marked by the presence of ferritin light chain (FLT) and ferritin heavy chain 1 (FTH1). Here, we observed that both ferritins were more abundant in the eBPD tissues and confirmed the higher abundance of the ferritins by Western blot assay (Fig. 5, *C*–*E*). Other ferroptosis-related proteins such as ALOX15 (arachidonate 15-lipoxygenase) that generates lipid mediators of inflammation and immunity (58), ATG7 (autophagy related 7), which promotes autophagy (59), and ISCA2 (iron-sulfur cluster assembly 2 protein), which influences ferroptosis through hypoxia-inducible factors (HIF) in other tissues (60) also trended up in patients with eBPD (Fig. 5*E*). To verify if iron deposition occurred in human BPD tissues, we performed Prussian blue staining and detected approximately three times as many positively stained cells in eBPD compared with hBPD or controls (Fig. 5, *A* and *B*). The majority of the eBPD Prussian blue stain appears in cells resembling macrophages in the alveolar space though there are also positive cells in the walls of the alveolar septa requiring further identification in future samples.

### Tight Junction Proteins Are Reduced in eBPD

**Figure 6.**
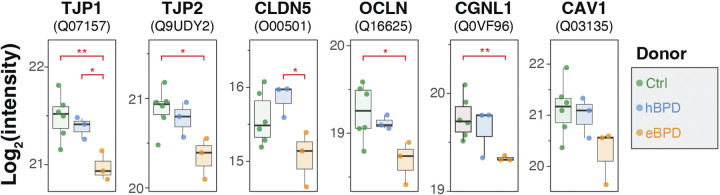
Combined dotplot and boxplot showing that tight junction proteins are less abundant in eBPD vs. Ctrl. Stars indicate *t* test *P* values (*P < 0.05,**P < 0.01) performed between two groups indicated by the red brackets. eBPD, established bronchopulmonary dysplasia.

EnrichR pathway analysis also predicted decreased cellular processes involved with barrier function, tight junctions, and cell-cell interactions (Fig. 1*E*). Here, we report decreases in several tight junction proteins including TJP1, TJP2, CLDN5, OCLN, and CGNL1 (Fig. 6).

## DISCUSSION

Bronchopulmonary dysplasia (BPD) describes a chronic lung disease of premature infants characterized by persistent oxygen requirement at or beyond 36 wk of corrected gestational age (CGA). BPD is a multifactorial disease resulting from premature birth, hyperoxia exposure, mechanical ventilation, inflammation, poor somatic and pulmonary growth, and other factors. In this study, taking advantage of the donor lung samples collected by the developmental lung molecular atlas program (LungMAP), we for the first time applied MPLEx proteomic analysis and a highly multiplexed immunofluorescent technique to human lungs to provide several valuable insights into the natural history of BPD. Our study is the first, to the best of our knowledge, to study in this detail a cohort of pediatric donor lungs, up to nearly 4 yr of age, affected with BPD. We undertook comparison of both hBPD and eBPD, classified by review of their histopathology and clinical status at death, to postnatal age-matched full-term donor controls to demonstrate proteomic abnormalities in severe BPD as well as to what extent the lung remains molecularly abnormal even when clinically healed after premature birth.

BPD has been most heavily studied in nonhuman primate and murine models of neonatal hyperoxia exposure but no model sufficiently or completely recapitulates the natural history of human BPD. The cellular and molecular biology occurring as preterm infants evolve from early, post surfactant-deficient respiratory distress syndrome into BPD near-term corrected age is not yet resolved. Frequently, if infants are able to survive the first 2 yr of life, infants with even severe BPD will have improved pulmonary function and no longer require respiratory support, clinically and pathologically termed “healed” BPD (hBPD). However, pulmonary function tests indicate persistent distal airway dysfunction, an obstructive or mixed obstructive/restrictive disorder (61). Studies into adulthood suggest “early aging” of lung function with signs of chronic obstructive pulmonary disease (COPD; 62). As shown in the current study, despite the resolution of clinical respiratory insufficiency, histopathology in young children with BPD history, remains abnormal most clearly characterized by enlarged, simplified alveolar structure.

Several observations in this study are consistent with previously reported findings in neonatal hyperoxia animal models. A recent study showed alveolar cell iron deposition in a murine neonatal hyperoxia exposure model (63). Ferroptosis describes a regulated process of cell death characterized by cellular iron accumulation, Fenton redox reactions, and lipid peroxidation (57) shown to contribute critically to the pathogenesis of chronic obstructive pulmonary disease and fibrosis (64). During ferroptosis, an unstable iron pool accumulates in association with ferritin light chain (FLT) and ferritin heavy chain 1 (FTH1) consistent with our identification of excessive intracellular iron and increased proteins, including ALOX15, that induce ferroptosis. Iron accumulation in alveolar macrophages accounts for the majority but does not appear to be the sole depot of iron by Prussian blue staining in these BPD lung cells, suggesting iron-induced cell death could contribute to the disease. Capillary leak and fluid retention in lung parenchyma are also hallmarks of active and chronic BPD. Hyperoxia models have documented reduced tight junction proteins including tight junction protein 1 (TJP1/ZO-1), claudin-4 (CLDN4), and occludin (OCLN) (65). This reduction was associated with a reduction of caveolin-1 (CAV1), whereas overexpression CAV1 in mice exposed to hyperoxia markedly antagonized the disappearance of tight junction proteins. In the proteomics dataset, we observed that many tight-junction proteins were reduced in abundance in eBPD including TJP1, TJP2, OCLN, and cingulin (CGN) with a trend toward decreased CAV1 (Fig. 6).

An important feature of human BPD is an elevation in pulmonary vascular resistance resulting in a state of pulmonary hypertension (PH) reported in meta-analysis as occurring in up to 2% of ELGANS without BPD and 39% of infants with severe BPD (66). BPD-PH is multifactorial and likely due to simplification of the pulmonary capillary vasculature as well as increased vascular smooth muscle. The latter is expected to respond to pulmonary vasodilators, whereas the former, related to alveolar simplification, may not. Hyperoxia and double-hit models recapitulate the PD-PH phenotype, which leads to heart failure and early death in experimental animals (67, 68). In humans, it is both an early neonatal and a recurrent late finding in BPD associated with increased morbidity and mortality and which can progress to lethal right ventricular heart failure. The proteomic dataset and our multiplexed immunofluorescence suggest reduction in capillary endothelial cell proteins including EDNRB a receptor for the endogenous vasodilator endothelin and marker for aerocyte type capillary cells. Also detected in this dataset is a reduction in FLT-1/VEGFR1, a receptor for vascular endothelial growth factor alpha (VEGF-A) that may contribute to a failure in capillary bed growth in BPD (69). Our data also suggest that excess ACTA2+ vascular smooth muscle occurs in eBPD and suggests that the vessels most affected are moderate in size matching bronchiolar airway levels. Future therapies to prevent or treat BPD-PH will depend, in part, on understanding the impact of smooth muscle presence, function, and contractility and how pulmonary vascular resistance is modulated in BPD.

One advantage of using proteomics is that it can identify changes in extracellular matrix (ECM) that are difficult to study using other methods. ECM is known to play a key role in lung development and its disruption has been well documented in BPD. ECM is in a constant state of turnover and though proteomics identifies the ECM at a snapshot in time, it indicates how the scaffold of the lung is altered by premature birth and NICU exposures. Some of our observed altered ECM disruptions in eBPD donor lungs have been replicated in animal models of BPD. Increased collagen 8 was associated with pulmonary diseases marked by vascular remodeling (70). Collagen 14 is suggested to play a role in pulmonary fibrotic processes (71, 72). Collagen 15 is a nonfibrillar collagen associated with the basement membranes of vascular endothelial cells in normal lungs (73, 74); however, aberrant expression of COL15A1, as detected in eBPD, has been observed in fibrotic lungs (74). Collagen 18 has been found localized around vasculature in normal lungs and its deposition was found increased in patients with pulmonary hypertension (75). In addition, several ECM proteins known to regulate angiogenesis such as ANXA2, ANXA3, and CSPG4 (chondroitin sulfate proteoglycan 4) were all decreased in eBPD, which may contribute to the observed loss or failure to repair endothelial cells. Furthermore, reduction in our eBPD matrisome of MMRN1, known to support the adhesion of platelets and neutrophils (76), typically found in close contact with endothelial cells and with a suggested role to maintain vessel integrity, may contribute to the disease (76). Annexins are Ca^2+^ and phospholipid binding proteins (77) and could play a role in fibrosis protection (78) and microvascular integrity (79). Similar to annexins, multiple S100 calcium-binding proteins were downregulated in eBPD and are involved in cell proliferation (S100A6), interactions with ANXA2 (S100A10), p53 and ECM regulation (S100A14), and insulin resistance (S100A16) (80). Other proteins found to be increased in eBPD, consistent with some rodent models, include fibrillin-2 (81), lysyl oxidase 2 (LOXL2) (82, 83), and MMP2 (matrix metalloproteinase 2), the latter despite prior reports that MMP2 decreases in tracheal aspirates of patients with BPD (84). The discrepancies between the experimental and human BPD findings included in this analysis may be attributable to analyses being done during oxygen exposure in rodents and relatively early in the course of BPD (humans) versus sampling of “older” infants with BPD. In addition, all of the eBPD donors were on chronic home ventilation and two out of three had superimposed pulmonary hypertension, which may not be adequately recapitulated by animal models.

Despite the novelty in performing proteomics on BPD donor lungs, there are limitations to this study. First, the small sample size, single analysis time point (end-stage BPD or death by nonrespiratory cause), and heterogeneity of BPD make statistical comparisons difficult. To improve the relevance and quality of molecular study of BPD, the cases studied are not the typical autopsy cases with prolonged postmortem anoxic warm ischemia times. These donor tissues are organ donations recovered from a transplantation network that maintain short warm ischemic times (<20 min) and cold ischemic protocols (i.e., flush with transplant buffer) to maintain tissue viability. Thus, these are highly unique preparations of tissue for the study of BPD and are limited in quantity and timing of disease by the gifting of organ donation in children. To address this shortcoming, the LungMAP HTC (human tissue core) continues to add BPD samples to the repository as they become available and with increasing samples will evaluate changes in BPD earlier and later in life and better address the heterogeneity and human variation of the disease. Second, the grading of BPD by severity of clinical respiratory failure and postmortem histopathology leads the most severe cases to “self-selecting” into the eBPD group, resulting in findings reminiscent of those reported by Northway though with less necrosis, metaplasia, cystic emphysema and peri-airway smooth muscle hypertrophy (85). The similarity of hBPD to age-matched control proteome, despite continued alveolar simplification, suggests that the balance of alveolar epithelial and capillary cell types is normalized with resolution of disease, and these unique findings add to our knowledge of BPD. Third, the proteomics was performed on whole lung tissue biopsy in a restricted site of distal lung which allows for comparisons but limited exploration of regional specificity for changes in protein abundance and may increase the probability of type I or type II statistical error. Additional analyses using laser capture microdissection with sampling from different tissue blocks are underway to identify large and small airway-specific changes in proteomics occurring in BPD. Finally, we did not find statistical differences in immune cell proteins in the proteomic analyses. However, it can be seen (Supplemental Fig. S2) that there is considerable heterogeneity in the presence of immune cells, including adaptive immune B and T cells organized in bronchus and bronchioalveolar lymphoid structures in the young lung with eBPD such that small biopsy and small ROI imaging are inadequate to reflect these cells well. Furthermore, in the current sampling, protein contributions from immune cells are relatively small compared with the parenchymal tissue. Immune cells are likely better studied using flow cytometry on dissociated cells if the cells can be captured. As technology progresses, MxIF panels can be expanded to combine immunophenotyping by flow or mass cytometry with larger field and three-dimensional MxIF to associate spatial localization of tissue-resident immune cells and predict immune-parenchymal interactions.

Finally, our classification of BPD is based largely on clinical presentation at time of death. The eBPD donors were ventilation- and/or cardiorespiratory medication-dependent, whereas two of the hBPD donors lacked these clinical features. The terms used to stratify BPD are not standard in the field and we are subtly making a recommendation that BPD be classified with finer resolution for research purposes. Those at 36 wk CGA, diagnosed clinically as “having BPD” are in a state of active evolution of the disease or “aeBPD.” Within their first corrected age year of life such babies will either symptomatically heal their BPD or they will remain ventilator and/or oxygen dependent. We suggest that if those infants remain technology dependent beyond 1 yr of age, their pathophysiology differs from those who have clinically recovered. Finally, our hBPD group, consistent with previous literature, although born with risks indistinguishable from the aeBPD or eBPD babies, have resolved their disease, but the lungs remain histologically abnormal, representing a probable risk factor for early adult respiratory decline. The nomenclature may change with time but there is a utility to distinguishing variations in the natural history of postprematurity respiratory disease.

This study highlights the need for future studies to focus on understanding and reversing the molecular changes associated with NICU exposures that can prevent the development of eBPD before they take hold. Equally important, however, is developing treatments to promote “healing” which may attenuate the eBPD phenotype and allow children to live longer, healthier lives.

## DATA AVAILABILITY

The data analysis code is publicly available at https://github.com/GeremyClair/Proteome_analysis_of_a_human_donor_cohort_to_study_bronchiopulmonary_displasia. 

## SUPPLEMENTAL DATA

10.6084/m9.figshare.22658710 Supplemental Tables S1 and S2, the Proteomics Dataset - Supplemental Data, and Supplemental Figs. S1–S4: https://doi.org/10.6084/m9.figshare.22658710.

## GRANTS

This work was supported by National Heart, Lung, and Blood Institute (NHLBI) Molecular Atlas of Lung Development Program Human Tissue Core (LungMAP HTC) grants U01HL122700 and U01HL148861 (to G.H. Deutsch, T.J. Mariani, R.S. Misra, P.J. Katzman, and G.S. Pryhuber), and U01 HL148860 (to J.N. Adkins and G.C. Clair). This work was also supported by NHLBI Career Development Award K08HL155491 (to A.M. Dylag). Part of this work was performed in the Environmental Molecular Sciences Laboratory (EMSL) from Pacific Northwest National Laboratory, a DOE Office of Science User Facility sponsored by the Office of Biological and Environmental Research and operated under Contract No. DE-AC05-76RL01830.

## DISCLOSURES

No conflicts of interest, financial or otherwise, are declared by the authors. 

## AUTHOR CONTRIBUTIONS

A.M.D., R.S.M., C.P., H.L.H., C.D., V.P., P.J.K., J.W., J.M.P., J.N.A., T.J.M., G.C.C., and G.S.P.. conceived and designed research; A.M.D., R.S.M., G.B., C.P., H.L.H., M.G.J., J.H., G.H.D., C.D., H.M.O., V.P., P.J.K., J.W., J.M.P., J.N.A., T.J.M., G.C.C., and G.S.P. performed experiments; A.M.D., R.S.M., G.B., C.P., H.L.H., M.G.J., J.H., G.H.D., C.D., H.M.O., V.P., P.J.K., J.W., J.M.P., J.N.A., T.J.M., G.C.C., and G.S.P. analyzed data; A.M.D., R.S.M., G.B., C.P., H.L.H., M.G.J., J.H., G.H.D., C.D., H.M.O., V.P., P.J.K., J.W., J.M.P., J.N.A., T.J.M., G.C.C., and G.S.P. interpreted results of experiments; A.M.D., C.P., H.L.H., M.G.J., J.H., H.M.O., J.W., J.M.P., G.C.C., and G.S.P. prepared figures; A.M.D., J.W., and G.C.C. drafted manuscript; A.M.D., R.S.M., G.B., C.P., H.L.H., M.G.J., J.H., G.H.D., C.D., H.M.O., V.P., P.J.K., J.W., J.M.P., J.N.A., T.J.M., G.C.C., and G.S.P. edited and revised manuscript; A.M.D., R.S.M., G.B., C.P., H.L.H., M.G.J., J.H., G.H.D., C.D., H.M.O., V.P., P.J.K., J.W., J.M.P., J.N.A., T.J.M., G.C.C., and G.S.P. approved final version of manuscript. 
